# Factors associated with essential newborn care practices among non-institutional births in urban Bangladesh: evidence from Bangladesh Urban Health Survey 2021

**DOI:** 10.1080/16549716.2024.2412152

**Published:** 2024-10-08

**Authors:** Shimlin Jahan Khanam, Mst. Fatema Begum, Md Badsha Alam, Md Awal Kabir, Md Nuruzzaman Khan

**Affiliations:** a Department of Population Science, Jatiya Kabi Kazi Nazrul Islam University, Mymensingh, Bangladesh; b Maternal and Child Health Division, International Centre for Diarrhoeal Disease Research, Bangladesh (icddr,b), Dhaka, Bangladesh; c Department of Social Work, Pabna University of Science and Technology, Pabna, Bangladesh; d Nossal Institute for Global Health, Melbourne School of Population and Global Health, The University of Melbourne, Melbourne, Australia

**Keywords:** Essential newborn care (ENC), non-institutional births, neonatal mortality, urban health, urban slum and non-slum, Bangladesh

## Abstract

**Background:**

Non-institutional births remain prevalent in low- and middle-income countries, associated with a majority of adverse maternal and child health outcomes, including maternal and child mortality. Ensuring essential newborn care (ENC) practices for these non-institutional births is crucial for reducing these adverse outcomes. This study aimed to identify the prevalence, and factors associated with the adoption of ENC practices among non-institutional births in urban Bangladesh.

**Methods:**

A total of 2,165 children’s data were analyzed, extracted from the 35,186 ever-married women interviewed in the 2021 Bangladesh Urban Health Survey. Six ENC components and their level (lowest/none, moderate, and highest) were considered as the outcome variables. Several socio-demographic factors were considered as the explanatory variables. Multivariate binary and multinomial logistic regression model were used to explore the association between outcome and explanatory variables.

**Results:**

Approximately 49% of all mothers reported practicing the highest level of ENC. Among the individual components, the highest adherence was observed for the use of a disinfected instrument to cut the umbilical cord (90%). The likelihood of adopting the highest level of ENC practices was higher among mothers with relatively higher education and wealth quintiles and lower among those residing in slum and other urban areas of city corporations compared to non-slum areas. Mothers living in the Khulna and Sylhet divisions had a lower likelihood of adopting the highest level of ENC practices.

**Conclusion:**

Awareness building programs are needed to educate the population, particularly mothers, about the importance of practicing ENC for improving maternal and child health outcomes.

## Paper context

- **Main findings**: The likelihood of utilizing the highest level of ENC practices was lower among mothers residing in slum and other urban areas of city corporations compared to those residing in non-slum areas.
- **Added knowledge**: Considering the limited studies on this specific topic especially for large-scale data of urban survey, the findings of this study contribute to a better understanding about essential newborns care (ENC) practices in urban areas of Bangladesh, particularly for urban-slum areas.
- **Global health impact for policy and action**: Our study contributes to be a better understanding of ENC patterns and which factors influence for ENC practices in adopting evidence-based policies and programmes for improving child health outcomes.

## Background

Child mortality remains a significant concern in low- and middle-income countries (LMICs), with an estimated 4.9 million under-five deaths occurring each year globally, translating to 13,400 deaths each day [1]. Approximately 2.3 million of these deaths occur during the first month of life (neonatal deaths), and 2.6 million between the ages of 1 month and 59 months [1,2]. Over 99% of these deaths occur in LMICs, with Asian and African countries representing the major share [3]. Among Asian countries, Bangladesh has the highest under-five mortality rate in South Asia, trailing only Pakistan and India [2,4]. These statistics highlight the challenge for LMICs, including Bangladesh, in achieving the Sustainable Development Goals’ targets for under-five mortality (25 per 1,000 live births) and neonatal mortality (12 per 1,000 live births) by 2030, as current rates are substantially higher [5].

Child mortality in LMICs are increasingly concentrated in the neonatal period, often caused by infectious diseases, preterm birth, intrapartum-related complications, pneumonia, birth asphyxia, prematurity, serious infections, drowning, and congenital anomalies [1]. Addressing these issues requires the utilization of maternal healthcare services and functioning healthcare systems. Approximately 40% of the total child mortality in LMICs and Bangladesh can be averted by ensuring Essential Newborn Care (ENC) – a set of interventions designed to promote the health and well-being of newborns during the crucial early stage of life [6]. These include immediate drying and wrapping of the newborn, delayed cord clamping, hygienic cord care, initiation of breastfeeding within the first hour, and thermal care [6]. These interventions foster optimal growth and development, establishing a strong foundation for a child’s future health [6,7]. Due to its significance, ENC is prioritized by international organizations, such as the World Health Organization, and national governments, with a target to ensure universal ENC practice [6].

The focus on ENC in LMICs and Bangladesh is inadequate, with nearly 54% of ENC utilization and substantial variations across ENC indicators [6,8,9]. Most ENC utilization occurs among the 65–72% of deliveries that take place in hospitals in LMICs and Bangladesh, with even higher rates in urban areas (76–80%) of these regions [10,11]. However, institutional delivery is lower in disadvantaged urban communities, including urban slums and among people with lower income and education [11,12]. These groups represent a growing proportion of the urban population, which currently constitutes 50% of the total population in LMICs [13,14]. Projections indicate that by 2050, around 68% will live in urban areas, with a significant rise in disadvantaged communities [13,14]. These estimates align with the current urban population in Bangladesh, which was 40.47% in 2023 and is expected to nearly double in the coming decades, with an increasing number of slums, poor, and uneducated individuals with lower incomes [15].

Research exploring ENC in urban areas is limited, characterized by small sample sizes and a restricted number of ENC indicators and maternal characteristics [8,9,16–19]. Additionally, existing studies often concentrate on specific ENC indicators, such as immediate initiation of breastfeeding and the utilization of boiled instruments to cut the umbilical cord [8,9,16,17]. This leads to a knowledge gap regarding ENC. Consequently, tracking the current state of ENC for evidence-based policy and program making is challenging. To address these challenges, we conducted this study to explore the current status of ENC practices in urban areas of Bangladesh and its associated socio-demographic factors.

## Methods

### Study setting and sampling procedure

The data for this study were extracted from the 2021 Bangladesh Urban Health Survey (BUHS), a nationally representative population-based household and community survey covering three urban domains (City Corporation slum, City Corporation non-slum, and other urban areas) across 11 city corporations [11]. The survey employed a stratified three-stage sampling technique to select sample households. In the first stage, 634 Mahallas (sub-wards of a union) were selected, comprising 450 from City Corporations and 184 from other urban areas, using proportional allocation based on the size of City Corporations and district municipalities/towns. In the second stage, 1,145 clusters (smallest geographical areas covering approximately 150 households, created by the Bangladesh Bureau of Statistics as part of the 2011 national census) were chosen from these Mahallas (300 from City Corporation slums, 600 from City Corporation non-slum areas, and 245 from other urban or district municipalities) using the probability-proportional-to-size method. In the third stage, 30 or 35 households (30 from City Corporation non-slum and other urban areas, 35 from City Corporation slums) were systematically selected from each cluster, resulting in 35,730 households for interviews (10,500 from City Corporation slums 17,880 from City Corporation non-slum areas, and 7,350 from other urban areas). Interviews were conducted in 34,972 households, yielding an inclusion rate of 97.88%. Among these households, there were 36,433 eligible women, of whom 35,186 were successfully interviewed, resulting in a response rate of over 96%. A detailed description of the sampling procedure has been published elsewhere [11].

### Study sample

We analyzed 2,165 children’s data from the original sample, consisting of all live-born home-delivered children in this study. Samples were selected based on the following inclusion criteria: (i) the most recent live birth of each mother in the three years preceding the survey, and (ii) information available on ENC practices for these births. The three-year restriction was imposed because the survey considered this time frame for collecting maternal healthcare services data.

### Outcome variables

The six individual components of ENC were the primary outcome variables of this study. The survey collected relevant data on these components by asking six different questions (Suplementary Table S1) with answer options of yes, no, or do not know. We used the National Neonatal Health Strategy and Guidelines for Bangladesh as a benchmark [11,20], which provides specific advice on the following ENC practices: (i) the use of boiled instruments to disinfect before cutting the umbilical cord, (ii) umbilical cord care involving not using any substances after it was cut and tied, (iii) drying the newborn within 5 min of birth, (iv) wrapping the newborn within 5 min of birth, (v) delayed bathing (bathing the newborn at least 72 h after birth), and (vi) initiation of breastfeeding within 1 h of delivery. In line with these recommendations, we recategorized all reported responses in binary form (yes, no) and considered them in the subsequent analyses for specific indicators. Additionally, we ran Multiple Correspondence Analysis (MCA), and based on this score, we created an adherence to ENC variable where a ≤25% score was considered lowest/none adherence to ENC, a 26.0–50% score was considered moderate adherence to ENC, and a >50% score was considered the highest adherence to ENC.

### Explanatory variables

Several socio-demographic variables were selected as explanatory factors through a comprehensive review of the relevant literature for Bangladesh and other LMICs [8,9,16–19]. These variables included mother’s age (≤19 years, 20–34 years, and ≥35 years), mothers’ education (no education, primary, secondary, and higher), mothers’ employment (yes, no), child’s gender (male, female), receipt of antenatal care (ANC) visit (<4 ANC and ≥4 ANC), religion (Muslim, Non-Muslim), wealth quintile (poorest, poorer, middle, richer, and richest), urban domain (city corporation slum, city corporation non-slum, and rest urban), and administrative division (Barishal, Chattogram, Dhaka, Khulna, Rajshahi, Rangpur, Sylhet, and Mymensingh).

### Statistical analysis

Descriptive statistics were used to summarize the characteristics of the respondents. A multivariate binary and multinomial logistic regression model were employed to identify the associations between explanatory and outcome variables. Multicollinearity was checked before running each model, and if detected, the relevant variable was excluded from the analysis. Sampling weight was considered in all analyses. The results of the multivariate binary logistic regression model are reported as adjusted odds ratios (aOR), while the results of the multinomial logistic regression model are presented as risk ratios (aRR) with their 95% confidence intervals (CIs). All statistical analyses were conducted using Stata version 17.0 (Stata Corporation, College Station, Texas, USA). This study was designed and reported following the Strengthening the Reporting of Observational Studies in Epidemiology (STROBE) guidelines (Supplementary Table S2).

## Results

### Background characteristics of the respondents

The background characteristics of the respondents are presented in Table 1. The majority of mothers (77.4%, 95% CI: 75.0–79.7) were in the age range of 20–34, with 12.5% (95% CI: 10.8–14.4) being ≤19 and 10.1% (95% CI: 8.3–12.1) ≥35 years. Around 42% (95% CI: 39.4–44.9) of the mothers had secondary education, and only 6.1% (95% CI: 4.7–7.7) had higher education. A notable proportion of mothers (83.0%, 95% CI: 81.0–85.6) were not employed, and 53.9% (95% CI: 51.5–56.3) of the children born were female. At least four ANC visits was received by 21.7% (95% CI: 18.9–24.9) of mothers, and 96% (95% CI: 93.8–97.4) of the mothers were Muslim. Nearly 32% (95% CI: 27.7–35.0) of the total mothers were included from the households of poorest quintile. The majority of mothers resided in city corporation slums (56.6%, 95% CI: 52.6–60.6). Around 42% (95% CI: 37.9–45.8) of the total mothers were included from the Chattogram division, followed by Dhaka (25.4%, 95% CI: 22.2–28.8) and Mymensingh (10.2%, 95% CI: 8.0–13.0) divisions.

**Table 1.** Background characteristics of the study participants, BUHS, 2021 (weighted sample = 2,165).

Characteristics	Percentage	95% CI
**Mother’s age in years, median (Q1-Q3)**		25.0 (21–30)
≤19	12.5	10.8-14.4
20-34	77.4	75.0-79.7
≥35	10.1	8.3-12.1
**Mother’s education**		
No education	10.8	9.2-12.5
Primary	41.1	38.3-43.8
Secondary	42.1	39.4-44.9
Higher	6.1	4.7-7.7
**Mother’s employment status**		
Yes	16.6	14.5-19.0
No	83.4	81.0-85.6
**Child’s gender**		
Male	46.1	43.7-48.5
Female	53.9	51.5-56.3
**Received antenatal care**		
<4 ANC	78.3	75.2-81.1
≥4 ANC	21.7	18.9-24.9
**Religion**		
Muslim	96.0	93.8-97.4
Non-Muslim	4.0	2.6-6.2
**Wealth quintile**		
Poorest	31.7	27.7-35.0
Poorer	25.7	22.8-28.9
Middle	23.8	21.2-26.6
Richer	14.0	11.9-16.4
Richest	4.8	3.6-6.3
**Urban domains**		
City corporation slum	56.6	52.6-60.6
City corporation non-slum	14.1	14.1-17.4
Rest urban+	29.3	25.6-33.2
**Division**		
Barishal	4.3	2.6-7.0
Chattogram	41.8	37.9-45.8
Dhaka	25.4	22.2-28.8
Khulna	5.0	3.9-6.3
Rajshahi	2.8	1.9-4.2
Rangpur	6.3	5.0-8.1
Sylhet	4.2	3.2-5.6
Mymensingh	10.2	8.0-13.0

^+^District municipalities and large towns.

### Prevalence of essential newborn care practices

**Figure 1.**
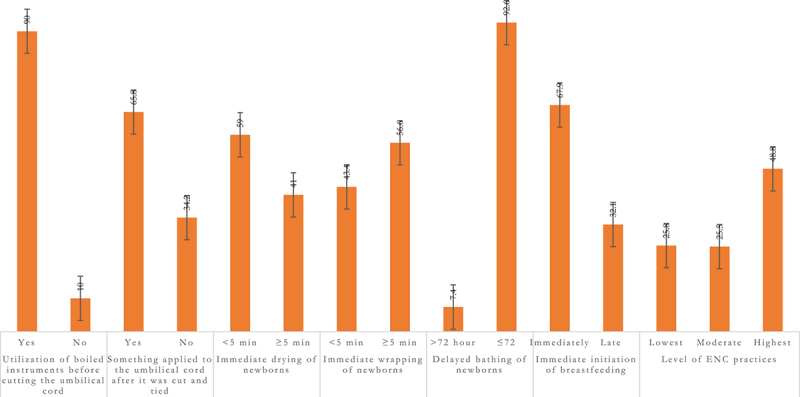
Percentage distribution of essential newborn care practice in urban Bangladesh (weighted sample= 2,165).

The percentage distribution of the six ENC practices is presented in Figure 1. Among the total mothers analysed, 92% reported using boiled water to disinfect the instruments to cut the umbilical cord, while approximately 34% mentioned not applying any substances to the umbilical cord after cutting. About 59% of mothers reported immediately drying the newborn, and 43.4% reported immediate wrapping of the newborn. Early initiation of breastfeeding was reported by 67.9% of the total mothers analysed. Only 7.4% of the total mothers reported delayed bathing. Around half of the total mothers reported utilizing the highest level of ENC practices in urban Bangladesh

### Variation of essential newborn care practice across explanatory variables

The percentage distribution of level of ENC practices and their different components are presented in Table 2. We observed variations in the prevalence of ENC practices across the considered explanatory variables. Mothers with comparatively higher education, engaged to formal employment, and a higher wealth quintile reported a highest level of ENC practice. We also noticed the differences in utilizing the highest level of ENC practices across urban domains, with the mothers living in non-slum areas of city corporation reporting 57% use of highest level of ENC compared to 49.2% and 43.8% among mothers living in slum and other urban areas of city corporations. In terms of administrative region, mothers residing in Khulna and Sylhet divisions had the lowest reported prevalence of utilizing higher level of ENC practices.

**Table 2.** Distribution of essential newborn care practices across explanatory variables, BUHS, 2021.

	Components of ENC practices						Level of ENC practices		
Characteristics	Utilization of boiled instruments before cutting the umbilical cord	Nothing applied to the umbilical cord after it was cut and tied	Immediate drying of newborns	Immediate wrapping of newborns	Delayed bathing of newborns	Early initiation of breastfeeding	Lowest/none	Moderate	Highest
**Mother’s age in years**									
≤19	89.9 (84.8–93.5)	41.1 (33.9–48.6)	54.1 (46.5–61.5)	41.0 (33.9–48.5)	13.1 (8.3–20.1)	68.4 (60.9–75.1)	24.3 (18.2–31.8)	23.0 (17.2–30.1)	52.7 (45.1–60.1)
20-34	89.9 (87.8–91.7)	33.1 (29.7–36.6)	60.2 (56.7–63.7)	44.1 (40.3–47.9)	7.0 (5.7–8.5)	68.6 (65.3–71.8)	26.2 (23.1–29.5)	26.3 (23.3–29.4)	47.6 (44.0–51.2)
≥35	90.3 (84.5–94.1)	34.7 (26.0–44.5)	55.1 (43.8–65.9)	41.8 (31.9–52.3)	3.4 (1.6–7.1)	61.4 (52.8–69.3)	24.4 (17.6–32.7)	22.5 (15.8–31.1)	53.1 (42.3–63.6)
**Mother’s education**									
No education	91.0 (85.7–94.5)	44.4 (36.5–52.6)	59.3 (51.4–66.8)	51.4 (43.7–59.1)	4.8 (2.7–8.4)	61.4 (54.0–68.3)	24.9 (19.1–31.7)	27.8 (21.4–35.2)	47.3 (39.7–55.1)
Primary	89.7 (86.6–92.1)	33.5 (28.9–38.4)	58.5 (53.6–63.2)	45.8 (40.9–50.8)	5.2 (3.6–7.4)	67.7 (63.5–71.6)	27.2 (23.4–31.5)	25.9 (22.1–30.1)	46.9 (42.1–51.6)
Secondary	89.8 (87.3–91.9)	33.7 (29.5–38.2)	58.9 (54.1–63.5)	40.0 (35.4–44.9)	9.7 (7.6–12.3)	67.7 (63.2–71.9)	24.0 (20.4–28.1)	25.4 (21.7–29.4)	50.6 (45.9–55.4)
Higher	90.9 (82.6–95.5)	24.7 (16.7–34.8)	62.0 (49.0–73.6)	37.0 (25.5–50.1)	10.6 (5.7–19.1)	81.4 (69.7–89.3)	29.5 (19.1–42.6)	19.3 (12.7–28.1)	51.3 (39.4–63.0)
**Mother’s employment status**									
Yes	91.4 (85.7–95.0)	37.2 (30.4–44.5)	62.7 (56.2–68.7)	48.4 (41.4–55.5)	3.7 (2.0–6.8)	63.4 (56.4–69.8)	25.9 (20.4–32.2)	28.0 (22.3–34.7)	46.1 (39.4–52.9)
No	89.7 (87.7–91.4)	33.6 (30.4–37.0)	58.2 (54.4–61.9)	42.5 (38.6–46.4)	8.0 (6.7–9.8)	68.8 (65.5–71.9)	25.7 (22.8–28.9)	25.0 (22.2–28.0)	49.3 (45.6–53.0)
**Child’s gender**									
Male	91.6 (89.5–93.3)	34.5 (30.5–38.8)	60.0 (55.7–64.1)	42.3 (37.0–45.8)	7.9 (5.9–10.6)	69.9 (66.1–73.4)	26.7 (23.1–30.7)	24.0 (20.7–27.7)	49.2 (44.8–53.6)
Female	88.5 (85.7–90.9)	34.0 (30.1–38.1)	58.0 (53.8–62.2)	45.3 (40.8–50.0)	6.9 (5.5–8.7)	66.1 (61.9–70.1)	24.9 (21.5–28.7)	26.7 (23.3–30.5)	48.4 (44.1–52.7)
**Received antenatal care**									
No and <4 ANC	88.9 (86.5–90.8)	35.5 (29.2–41.2)	57.8 (54.3–61.3)	43.2 (39.3–47.1)	7.1 (5.7–8.8)	68.6 (65.2–71.7)	25.6 (22.6–28.9)	24.9 (22.0–28.0)	49.6 (45.9–53.2)
≥4 ANC	93.9 (90.7–96.0)	29.6 (23.9–36.0)	63.0 (54.5–70.8)	44.5 (37.1–52.2)	8.4 (5.8–12.0)	65.4 (58.9–71.3)	26.4 (21.2–32.4)	27.7 (22.3–33.8)	45.9 (38.1–53.9)
**Religion**									
Muslim	89.9 (87.9–91.6)	34.1 (30.8–37.6)	58.9 (55.4–62.3)	43.3 (39.5–47.1)	7.2 (6.0–8.6)	68.1 (64.9–71.0)	25.6 (22.7–28.7)	25.7 (23.0–28.6)	48.7 (45.2–52.3)
Non-Muslim	91.6 (81.6–96.4)	37.1 (25.4–50.6)	59.8 (44.1–73.6)	48.0 (35.9–60.4)	11.8 (5.2–24.6)	63.3 (47.8–76.5)	30.5 (20.1–43.3)	20.4 (11.7–33.0)	49.2 (36.3–62.2)
**Wealth quintile**									
Poorest	85.8 (81.4–89.3)	36.9 (31.3–42.9)	59.3 (53.4–65.0)	44.5 (38.0–51.1)	8.8 (6.4–11.9)	74.0 (68.9–78.6)	28.3 (23.0–34.3)	21.8 (17.9–26.2)	49.9 (43.9–56.0)
Poorer	91.8 (88.3–94.2)	36.6 (31.1–42.4)	60.2 (55.0–65.2)	43.3 (37.7–49.1)	5.3 (3.5–7.9)	66.8 (62.1–71.2)	24.8 (20.6–29.5)	27.7 (23.3–32.7)	47.5 (42.5–52.5)
Middle	92.1 (88.8–94.5)	30.1 (25.0–35.8)	58.3 (52.3–64.1)	43.9 (37.7–50.4)	6.4 (4.1–9.6)	63.1 (57.4–68.4)	26.2 (21.2–31.9)	25.6 (20.7–31.2)	48.2 (41.9–57.2)
Richer	92.0 (87.8–94.9)	34.0 (27.2–41.5)	57.4 (48.2–66.0)	44.5 (36.4–52.9)	8.2 (5.3–12.5)	59.7 (50.3–68.2)	22.0 (16.9–28.2)	29.1 (23.4–36.1)	48.8 (40.6–57.2)
Richest	91.1 (82.4–95.7)	24.8 (15.3–37.7)	57.5 (43.1–70.7)	32.0 (21.7–44.4)	11.9 (6.6–20.5)	80.7 (70.8–87.8)	23.1 (14.3–35.0)	26.7 (15.8–41.5)	50.2 (36.3–64.1)
**Urban domains**									
City corporation slum	90.5 (88.3–92.4)	37.8 (33.5–42.3)	58.6 (54.7–62.3)	44.8 (40.2–49.4)	5.8 (4.5–7.5)	66.2 (62.5–69.8)	23.6 (20.3–27.1)	27.3 (23.9–30.9)	49.2 (45.1–53.3)
City corporation non-slum	91.9 (85.2–95.7)	34.8 (24.8–46.4)	50.7 (39.2–62.2)	31.8 (23.1–42.0)	7.4 (3.8–14.0)	64.3 (53.6–73.7)	20.9 (14.0–30.0)	21.8 (14.4–31.5)	57.3 (45.7–68.2)
Rest urban^+^	88.0 (83.6–92.3)	27.0 (22.5–32.1)	63.7 (57.1–69.8)	46.5 (39.8–53.5)	10.4 (7.9–13.5)	72.8 (67.3–77.7)	32.4 (26.7-38.7-38.7)	23.8 (19.2–29.2)	43.8 (37.7–50.1)
**Division**									
Barishal	94.7 (86.2–98.1)	33.1 (25.7–41.4)	87.0 (74.4–93.9)	20.1 (5.6–51.5)	8.9 (3.1–22.8)	68.9 (49.5–83.4)	21.7 (8.6–45.1)	42.1 (25.4–60.8)	36.2 (28.6–44.6)
Chattogram	90.8 (87.3–93.4)	31.6 (26.4–37.2)	57.7 (51.9–63.3)	48.7 (42.8–54.8)	7.4 (5.6–9.9)	64.7 (59.8–69.3)	29.6 (24.7–35.0)	23.7 (19.5–28.4)	46.7 (40.9–52.6)
Dhaka	89.2 (85.3–92.1)	36.8 (30.4–43.8)	57.0 (50.7–63.1)	41.9 (35.4–48.7)	6.5 (4.4–9.5)	69.6 (64.2–74.5)	20.2 (15.4–26.0)	28.0 (22.7–34.1)	51.8 (44.9–68.7)
Khulna	87.3 (77.5–93.2)	41.0 (33.5–48.9)	74.3 (64.2–82.3)	61.0 (48.3–72.4)	13.3 (7.0–24.0)	82.3 (68.6–90.8)	34.3 (25.6–44.3)	33.5 (25.3–42.8)	32.2 (23.1–42.8)
Rajshahi	84.5 (70.1–92.7)	34.3 (22.6–48.4)	63.0 (44.1–78.6)	38.1 (18.4–62.7)	16.2 (8.4–28.9)	71.7 (61.6–80.0)	25.9 (14.6–41.6)	19.3 (9.5–35.4)	54.8 (32.9–75.0)
Rangpur	87.3 (78.0–93.1)	39.0 (25.2–54.8)	50.1 (38.9–61.3)	43.4 (27.9–60.3)	2.6 (0.8–8.3)	67.5 (51.8–80.1)	18.1 (9.9–30.7)	22.3 (13.8–33.9)	59.7 (46.1–71.9)
Sylhet	93.9 (86.0–97.5)	24.3 (15.4–36.2)	84.0 (67.9–92.9)	56.3 (33.6–76.6)	4.5 (1.6–12.4)	78.0 (67.0–86.1)	43.3 (31.1–56.3)	35.6 (22.6–51.1)	21.2 (10.4–38.2)
Mymensingh	89.4 (82.4–93.8)	37.0 (27.2–48.1)	43.8 (33.5–54.7)	24.4 (14.8–37.5)	7.5 (4.5–12.4)	63.4 (51.5–73.9)	19.0 (13.4–26.3)	15.2 (9.4–23.6)	65.8 (54.7–75.4)

Presented as row percentages. ^+^District municipalities and large towns.

### Factors associated with the levels and each component of essential newborn care (ENC) practices in urban Bangladesh

The associations between ENC practices and explanatory variables are presented in Table 3. We found likelihoods of moderate and higher levels of ENC practices increased with the mothers’ increased age and education levels, as compared to the mothers with comparatively lower age and lower education. Employed mothers had a lower likelihood of reporting higher level of ENC practice as compared to unemployed mothers. Additionally, as compared to the mothers resided in the poorest households, the likelihoods of moderate and higher levels of ENC practices were found higher among mothers in the households with higher wealth quintiles. Furthermore, we found a 55% lower likelihood (RR, 0.45, 95% CI, 0.31–0.66) of higher levels of ENC practices among mothers living in other urban areas (district municipalities and large towns) compared as compared to the mothers resided in rest urban areas. Mothers residing in the Khulna and Sylhet divisions reported lower likelihoods of moderate and higher level of ENC practices as compared to those residing in the Barishal division.

**Table 3.** Results from multinomial logistic regression model to assess the association between the level of ENC practices and explanatory variables, Bangladesh, BUHS, 2021.

Characteristics	Moderate vs Lowest/none		Highest vs Lowest/none	
Mother’s age in years	aRR	95% CI	aRR	95% CI
≤19 (ref)				
20-34	1.13	0.77-1.65	0.94	0.68-1.30
≥35	1.07	0.62-1.83	1.14	0.72-1.80
**Mother’s education**				
No education (ref)				
Primary	0.85	0.57-1.29	0.97	0.67-1.40
Secondary	0.97	0.63-1.49	1.22	0.83-1.80
Higher	0.55	0.28-1.08	1.19	0.67-2.09
**Mother’s employment status**				
No (ref)				
Yes	1.01	0.73-1.40	0.87	0.65-1.18
**Child’s gender**				
Male (ref)				
Female	1.21	0.95-1.54	1.03	0.83-1.27
**Received antenatal care**				
<4 ANC (ref)				
≥4 ANC	1.03	0.76-1.39	0.88	0.67-1.15
**Religion**				
Non-Muslim (ref)				
Muslim	1.44	0.77-2.70	1.32	0.78-2.22
**Wealth quintile**				
Poorest (ref)				
Poorer	1.32	0.94-1.85	1.11	0.82-1.50
Middle	1.13	0.78-1.65	0.98	0.71-1.35
Richer	**1.60**	**1.04-2.47**	1.17	0.80-1.72
Richest	1.59	0.82-3.08	1.23	0.68-2.20
**Urban domains**				
City corporation non-slum (ref)				
City corporation slum	1.27	0.84-1.90	0.88	0.62-1.24
Rest urban^+^	0.85	0.54-1.33	**0.45**	**0.31-0.66**
**Division**				
Barishal (ref)				
Chattogram	**0.32**	**0.18-0.58**	0.84	0.46-1.53
Dhaka	0.55	0.29-1.02	1.28	0.69-2.39
Khulna	**0.38**	**0.18-0.80**	**0.44**	**0.21-0.94**
Rajshahi	0.40	0.16-1.02	1.48	0.65-3.40
Rangpur	0.61	0.28-1.32	1.98	0.95-4.11
Sylhet	**0.36**	**0.17-0.75**	**0.27**	**0.12-0.61**
Mymensingh	**0.41**	**0.20-0.85**	2.20	1.13-4.30

**a**RR= Adjusted Risk Ratios. Ref= Reference group. ^+^District municipalities and large towns.

We also explored each component of ENC practices across the considered explanatory variables and the relevant results are presented in Table 4. The utilization of boiled instruments for cutting the umbilical cord increased with rising wealth quintile from poorest wealth quintile. The relevant likelihood was found lower in the Dhaka division as compared to the Barishal division. Mothers living in city corporation slums (aOR, 1.81, 95% CI, 1.42–2.32) and non-slum areas (aOR, 1.77, 95% CI, 1.28–2.44) reported a higher likelihoods of not applying anything to the umbilical cord in contrast to the rest of the urban areas. Conversely, mothers in households of middle (aOR, 0.60, 95% CI, 0.45–0.79) and richest (aOR, 0.56, 95% CI, 0.33–0.95) wealth quintile were less likely to apply anything to the umbilical cord compared to the mothers in households of poorest wealth quintile. The likelihoods of immediately drying newborns were found higher among employed mothers and who received four or more antenatal care visits. However, the relevant likelihoods were found lower among mothers from city corporation non-slums, and mothers from Chattogram, Dhaka, Rajshahi, Rangpur, and Mymensingh divisions. The likelihoods of immediate wrapping of newborns was found lower among mothers in city corporation slums and non-slums areas as compared to the mothers resided in rest of urban areas. However, we found the corresponding likelihoods were higher among mothers in the Chattogram, Dhaka, Khulna, Rajshahi, Sylhet, and Rangpur divisions as compared to the mothers resided in the Barishal division. We found lower likelihoods of delayed bathing of newborns among mothers aged 20–34 (aOR, 0.49, 95% CI, 0.32–0.75) and ≥ 35 (aOR, 0.24, 95% CI, 0.10–0.55) as compared to mothers aged ≤19 years. The likelihoods of early initiating breastfeeding were found higher among mothers with higher education (aOR, 2.57, 95% CI, 1.47–4.48)) and those residing in the Khulna division (aOR, 2.58, 95% CI, 1.30–5.12) compared to illiterate mothers and those in the Barishal division, respectively. Conversely, the likelihoods of early initiation of breastfeeding were found lower among mothers whose index child was female, mothers from comparatively higher wealth quintiles, and mothers residing in the Mymensingh division.

**Table 4.** Results from multivariate binary logistic regression model to assess the factors associated with essential newborn care (ENC) practices in urban Bangladesh, 2021.

	Components of ENC practices					
	Utilization of boiled instruments before cutting the umbilical cord	Nothing applied to the umbilical cord after it was cut and tied	Immediate drying of newborns	Immediate wrapping of newborns	Delayed bathing of newborns	Early initiation of breastfeeding
Characteristics	aOR (95% CI)	aOR (95% CI)	aOR (95% CI)	aOR (95% CI)	aOR (95% CI)	aOR (95% CI)
**Mother’s age in years**						
≤19 (ref)	1.00	1.00	1.00	1.00	1.00	1.00
20-34	1.03 (0.66–1.59)	0.73 (0.55–0.95)**	1.24 (0.94–1.60)	1.04 (0.79–1.36)	0.49 (0.32–0.75)***	0.97 (0.73–1.29)
≥35	1.10 (0.59–2.05)	0.67 (0.46–0.99)**	1.02 (0.70–1.50)	0.85 (0.58–1.25)	0.24 (0.10–0.55)***	0.74 (0.50–1.10)
**Mother’s education**						
No education (ref)	1.00	1.00	1.00	1.00	1.00	1.00
Primary	0.89 (0.53–1.48)		0.92 (0.68–1.26)	0.72 (0.53–0.98)**	0.89 (0.45–1.77)	1.26 (0.92–1.72)
Secondary	0.89 (0.52–1.51)	0.64 (0.47–0.86)***	0.92 (0.67–1.27)	0.59 (0.43–0.81)***	1.34 (0.69–2.63)	1.25 (0.91–1.73)
Higher	0.87 (0.39–1.95)	0.55 (0.33–0.91)***	0.83 (0.51–1.36)	0.54 (0.33–0.88)**	1.23 (0.51–2.99)	2.57 (1.47–4.48)***
**Mother’s employment status**						
No (ref)	1.00	1.00	1.00	1.00	1.00	1.00
Yes	1.14 (0.75–1.75)	1.13 (0.88–1.46)	1.30 (1.03–1.67)**	1.21 (0.95–1.54)	0.60 (0.33–1.10)	0.87 (0.68–1.12)
**Child’s gender**						
Male (ref)	1.00	1.00	1.00	1.00	1.00	1.00
Female	0.73 (0.55–0.97)**	0.97 (0.80–1.18)	0.95 (0.80–1.14)	1.18 (0.99–1.41)	0.86 (0.62–1.20)	0.83 (0.68–0.99)**
**Received antenatal care**						
<4 ANC (ref)	1.00	1.00	1.00	1.00	1.00	1.00
≥4 ANC	1.82 (1.19–2.78)***	0.79 (0.63–1.00)	1.24 (0.99–1.56)	1.16 (0.93–1.46)	1.03 (0.68–1.54)	0.81 (0.64–1.03)
**Religion**						
Non-Muslim (ref)	1.00	1.00	1.00	1.00	1.00	1.00
Muslim	0.82 (0.37–1.80)	0.91 (0.48–1.44)	0.92 (0.58–1.46)	0.72 (0.46–1.13)	0.49 (0.25–0.9)**	1.26 (0.79–2.01)
**Wealth quintile**						
Poorest (ref)	1.00	1.00	1.00	1.00	1.00	1.00
Poorer	1.88 (1.25–2.82)***	0.85 (0.65–1.09)	0.93 (0.72–1.21)	0.82 (0.64–1.06)	0.65 (0.40–1.06)	0.62 (0.47–0.82)***
Middle	1.84 (1.18–2.87)***	0.60 (0.45–0.79)***	0.92 (0.70–1.21)	0.89 (0.68–1.18)	0.89 (0.54–1.48)	0.56 (0.42–0.75)***
Richer	1.76 (1.04–2.98)**	0.76 (0.55–1.06)	0.92 (0.67–1.27)	0.93 (0.68–1.28)	1.22 (0.70–2.14)	0.50 (0.39–0.69)***
Richest	1.36 (0.62–2.97)	0.56 (0.33–0.95)***	0.87 (0.54–1.40)	0.62 (0.38–1.01)	1.63 (0.77–3.45)	1.26 (0.71–2.21)
**Urban domains**						
Rest urban^+^ (ref)	1.00	1.00	1.00		1.00	1.00
City corporation slum	1.02 (0.70–1.47)	1.81 (1.42–2.32)***	0.72 (0.57–0.92)***	0.68 (0.54–0.86)***	0.53 (0.35–0.81)***	0.91 (0.71–1.17)
City corporation non-slum	1.28 (0.76–2.15)	1.77 (1.28–2.44)***	0.54 (0.40–0.73)***	0.47 (0.34–0.64)***	0.59 (0.35–1.05)	0.72 (0.52–1.01)
**Division**						
Barishal (ref)	1.00	1.00	1.00	1.00	1.00	1.00
Chattogram	0.50 (0.19–1.30)	0.82 (0.50–1.34)	0.21 (0.11–0.40)***	3.94 (2.29–6.80)***	1.20 (0.54–2.65)	1.11 (0.68–1.80)
Dhaka	0.38 (0.14–0.99)**	1.04 (0.64–1.70)	0.22 (0.12–0.42)***	3.24 (1.85–5.67)***	1.19 (0.51–2.74)	1.49 (0.89–2.47)
Khulna	0.35 (0.12–1.05)	1.12 (0.61–2.04)	0.45 (0.21–0.97)**	6.90 (3.56–13.36)***	2.75 (1.05–7.18)**	2.58 (1.30–5.12)**
Rajshahi	0.35 (0.11–1.12)	1.08 (0.54–2.18)	0.23 (0.10–0.51)***	2.18 (1.04–4.55)**	2.26 (0.82–6.25)	1.09 (0.53–2.45)
Rangpur	0.51 (0.18–1.47)	1.07 (0.60–1.89)	0.15 (0.08–0.31)***	2.86 (1.53–5.35)***	0.32 (0.09–1.18)	0.90 (0.50–1.62)
Sylhet	0.72 (0.20–2.59)	0.61 (0.31–1.18)	0.83 (0.36–1.93)	5.44 (2.75–10.76)***	0.74 (0.21–2.61)	2.05 (1.03–4.07)**
Mymensingh	0.55 (0.20–1.54)	1.02 (0.60–1.74)	0.11 (0.06–0.22)***	1.18 (0.64–2.22)	1.03 (0.42–2.54)	0.80 (0.47–1.38)**

***p<0.01; **<0.05. 95% CI: 95% Confidence intervals. Ref: Reference group. ^+^District municipalities and large towns.

## Discussion

The objective of this study was to investigate the current status of ENC practices among home-born children in urban areas of Bangladesh and to identify the factors associated with ENC practices. We found approximately 49% of the total mothers reported the highest level of ENC practices. Among the different components of ENC practices, a higher percentage of utilizing boiled instruments was reported by 90% of the total mothers analyzed in the study, followed by immediate initiation of breastfeeding (67.9%). Delayed bathing of newborns was the found as the lowest reported ENC indicators (7.4%). However, this reported prevalence varied across socio-demographic characteristics, with mothers who were educated and belonged to higher wealth quintiles reporting higher prevalence. Similarly, the likelihoods of achieving the highest and moderate levels of ENC practices differed based on various characteristics. Significant factors of practicing the highest and moderate ENC included increased age of mothers, higher educational levels, and mothers from household of higher wealth quintiles. Conversely, employed mothers, other urban residents, and those living in Khulna and Sylhet divisions reported lower likelihoods of practicing the highest and moderate levels of ENC. These findings collectively suggest that urban areas in Bangladesh still face challenges in ensuring safe ENC practices.

This study revealed that only half of the mothers who delivered their last child at home reported practicing higher levels of ENC indicators, a proportion significantly lower than the WHO recommendation for universal access to ENC practices. However, comparing our findings with studies from Bangladesh and other LMICs is challenging, as these studies often focused on specific segments of ENC indicators, primarily early initiation of breastfeeding, whereas our findings considered all ENC indicators [6,8,9,18]. If we focus on particular indicators, our results are comparable with those of previous studies [6,8,9,18]. The reasons for this inadequacy can be descried by a range of factors. However, rapid and unplanned urbanization, currently faced by Bangladesh and other LMICs, emerges as a major contributor, leading to increased population density and poor healthcare resources [21,22]. Despite the proximity of urban centers to medical services, challenges persist due to overcrowded clinics, transportation issues, and disparities in healthcare distribution [23]. Socioeconomic inequalities in urban settings, with marginalized populations facing financial constraints and limited education, hinder access to essential maternal and newborn health services [24,25]. Moreover, urban areas exhibit diverse cultural landscapes, contributing to variations in healthcare-seeking behaviors and adherence to ENC guidelines [26,27].

An interesting observation in this study is that individual ENC indicator usage levels vary across socio-demographic characteristics, with the same respondents reporting use in one indicator while do not use in others. This observation is unusual for LMICs and Bangladesh, where previous studies reported a constant patterning, such as educated mothers always being better off in utilizing healthcare services compared to their uneducated counterparts [28–30]. This clearly indicates the influence of norms and traditions over ENC practices, along with the advancement of awareness [31]. For instance, their combined influence led to almost universal use of boiled instruments to cut the umbilical cord due to increasing awareness, while there was very minimal prevalence of delayed bathing of newborns [8]. Clearly, this indicates the need for comprehensive education, which itself was found to be an influential determinant for a few specific indicators of ENC in previous studies [24,28]. Addressing this complex interplay necessitates targeted interventions focusing on improving healthcare infrastructure, reducing socioeconomic disparities, and promoting culturally sensitive practices to enhance ENC in urban areas of Bangladesh [32,33].

The observed differences in the prevalence of ENC practices across various types of urban areas in Bangladesh can be attributed to a combination of factors. Firstly, urban areas encompass diverse socioeconomic landscapes, ranging from affluent neighborhoods to impoverished slums [34]. These variations in economic status influence access to healthcare services, awareness levels, and adherence to recommended practices [31]. City corporations, being administrative and economic centers, have better healthcare infrastructure and resources compared to other urban areas, contributing to more favorable ENC practices [35]. Secondly, within city corporations, the disparities between slums and non-slum areas play a crucial role [11]. Slum environments often face challenges such as overcrowding, inadequate sanitation, and limited access to healthcare facilities [36]. These conditions can impact the ability of residents to adopt and implement recommended ENC practices due to resource constraints and a lack of awareness [37]. Thirdly, cultural and educational differences among urban populations can influence healthcare-seeking behaviors and adherence to ENC guidelines [38]. Factors such as education levels, awareness campaigns, and community norms play a significant role in shaping maternal and newborn care practices [8]. Variations in these factors across different urban settings contribute to the observed differences in ENC prevalence [11]. Overall, these interlinkages suggest a need for increasing awareness regarding existing cultural norms and traditions. Improving healthcare infrastructure and promoting culturally sensitive practices for the optimal use of ENC are also crucial.

This study provides a robust exploration of the current status of ENC and examines the factors influencing ENC practices among non-institutional births in Bangladesh. The nationwide representation of the survey ensures comprehensive insights, encompassing diverse urban areas. The inclusion of a substantial sample size enhances statistical power. Moreover, the comprehensive evaluation of various ENC indicators and the use of sophisticated statistical methods provide a nuanced understanding of the multifaceted nature of non-institutional births. Together, these elements facilitate a more reliable exploration of the factors associated with ENC practices, which can be utilized for national-level policies and programs. However, despite these strengths, the study has inherent limitations. The cross-sectional design of the survey we analyzed restricts our ability to establish causal relationships between identified factors and ENC practices. The reliance on self-reported data introduces the potential for recall bias and social desirability bias. While the study acknowledged the influence of cultural norms and traditions on ENC practices, the survey’s constraints may limit a comprehensive exploration of these cultural dynamics. The exclusive focus on non-institutional births narrows the scope of generalizability, overlooking insights into ENC practices within healthcare facilities. Additionally, the data are extracted from the 2021 survey, and the study may not capture potential changes in socio-economic or healthcare policies post-dating this period. However, instead of these limitations, the nature of this study positions it to inform relevant policies and programs in Bangladesh.

## Conclusion

This study found that around half of the mothers do not practice the highest levels of ENC, with significant variations in utilization across several ENC indicators. The practice was notably lower among mothers with disadvantaged socio-demographic characteristics, including lower education and poorer wealth quintiles. Country-level policies and programs to increase awareness about ENC practices are important, with a primary focus on mothers with disadvantaged characteristics.

## Supplementary Material

Supplementary table 2.doc

Supplementary table 1.docx

## Abbreviations

LMICs= Low- and middle-income countries, BUHS= Bangladesh Urban Health Survey, WHO= World Health Organizations, SDGs= Sustainable Development Goals, PSU= Primary sampling Unit, aOR= adjusted Odds Ratio, CI= Confidence Interval.

## References

[1] UNICEF. Under-five mortality, 2022. UNICEF data: monitoring the situation of children and women. 2022 [accessed 2024 June12]. https://data.unicef.org/topic/child-survival/under-five-mortality.

[2] Rahman AE, Hossain AT, Siddique AB, et al. Child mortality in Bangladesh–why, when, where and how? A national survey-based analysis. J Glob Health. 2021;11:11. doi: 10.7189/jogh.11.04052 PMC8442576 34552721

[3] Sharrow D, Hug L, You D, et al. Global, regional, and national trends in under-5 mortality between 1990 and 2019 with scenario-based projections until 2030: a systematic analysis by the UN inter-agency group for child mortality estimation. The Lancet Global Health. 2022;10:e195–11. doi: 10.1016/S2214-109X(21)00515-5 35063111 PMC8789561

[4] Villavicencio F, Perin J, Eilerts-Spinelli H, et al. Global, regional, and national causes of death in children and adolescents younger than 20 years: an open data portal with estimates for 2000–21. The Lancet Global Health. 2024;12:e16–e7. doi: 10.1016/S2214-109X(23)00496-5 37898143

[5] Commission BP. Sustainable development goals: Bangladesh progress report 2020. Bangladesh Planning Commission: Dhaka, Bangladesh; 2020.

[6] Essential Newborn Care. [database on the Internet] 2022.

[7] Britto PR. Early moments matter for every child. Chile: ERIC; 2017. https://eric.ed.gov/?id=ED589952

[8] Jamee AR, Kumar Sen K, Bari W. Skilled maternal healthcare and good essential newborn care practice in rural Bangladesh: a cross‐sectional study. Health Sci Rep. 2022;5:e791. doi: 10.1002/hsr2.791 35989946 PMC9382035

[9] Kim ET, Singh K. The state of essential newborn care by delivery location in Bangladesh. Matern Child Health J. 2017;21:2078–2085. doi: 10.1007/s10995-017-2319-7 28712021 PMC6714055

[10] Hernández-Vásquez A, Chacón-Torrico H, Bendezu-Quispe G. Prevalence of home birth among 880,345 women in 67 low-and middle-income countries: a meta-analysis of demographic and health surveys. SSM-Popul Health. 2021;16:100955. doi: 10.1016/j.ssmph.2021.100955 34805477 PMC8581368

[11] National Institute of Population Research and Training (NIPORT). Urban health survey 2021. Dhaka: National Institute of Population Research and Training (NIPORT); 2022. https://www.bing.com/ck/a?!&&p=0c8ec10d9e6b06ddJmltdHM9MTcyODAwMDAwMCZpZ3VpZD0xYzFhNDg1OC04OTg4LTY2YTYtMDQ1NS01Y2M1ODgxMzY3MGUmaW5zaWQ9NTE4Mw&ptn=3&ver=2&hsh=3&fclid=1c1a4858-8988-66a6-0455-5cc58813670e&psq=National+Institute+of+Population+Research+and+Training+(NIPORT).+Urban+health+survey+2021.&u=a1aHR0cHM6Ly9uaXBvcnQucG9ydGFsLmdvdi5iZC9zaXRlcy9kZWZhdWx0L2ZpbGVzL2ZpbGVzL25pcG9ydC5wb3J0YWwuZ292LmJkL3B1YmxpY2F0aW9ucy82NmM0YWNjZF80YzZhXzRhYWJfOTAyY183ODFhNzdhYTg3NjgvMjAyMy0wMS0zMC0wNi0wNC05MzEwZmMxZjM5MDJjZGQwMzg4NDEyNGM2MDBkZGM4ZC5wZGYjOn46dGV4dD1UaGlzIHJlcG9ydCBwcmVzZW50cyB0aGUgZmluZGluZ3Mgb2YgdGhlIFVyYmFuIEhlYWx0aCBTdXJ2ZXk&ntb=1

[12] Khan MN, Kumar P, Rahman MM, et al. Inequalities in utilization of maternal reproductive health care services in urban Bangladesh: a population-based study. Sage Open. 2020;10:2158244020914394. doi: 10.1177/2158244020914394

[13] Kundu D, Pandey AK. World urbanisation: trends and patterns. In: Kundu D, Sietchiping R, Kinyanjui M, editors. Developing national urban policies. Singapore: Springer; 2020. doi: 10.1007/978-981-15-3738-7_2

[14] Gu D, Andreev K, Dupre ME. Major trends in population growth around the world. China CDC Weekly. 2021;3:604. doi: 10.46234/ccdcw2021.160 34594946 PMC8393076

[15] Pettit C, Wentz E, Randolph B, et al. Tackling the challenge of growing cities: an informed urbanisation approach. In: Hawken S, Han H, Pettit C, editors. Open Cities | Open Data. Singapore: Palgrave Macmillan; 2020. doi: 10.1007/978-981-13-6605-5_9

[16] Saaka M, Ali F, Vuu F. Prevalence and determinants of essential newborn care practices in the Lawra District of Ghana. BMC Pediatr. 2018;18:1–12. doi: 10.1186/s12887-018-1145-4 29793543 PMC5968597

[17] Reinders S, Blas MM, Neuman M, et al. Prevalence of essential newborn care in home and facility births in the Peruvian Amazon: analysis of census data from programme evaluation in three remote districts of the Loreto region. The Lancet Reg Health–Americas. 2023;18:18. doi: 10.1016/j.lana.2022.100404 PMC9950545 36844009

[18] Abebe M, Kejela G, Chego M, et al. Essential newborn care practices and associated factors among home delivered mothers in Guto Gida District, east wollega zone. PloS Global Public Health. 2023;3:e0001469. doi: 10.1371/journal.pgph.0001469 36963077 PMC10021559

[19] Efa BW, Berhanie E, Desta KW, et al. Essential new-born care practices and associated factors among post natal mothers in Nekemte City, Western Ethiopia. PLOS ONE. 2020;15:e0231354. doi: 10.1371/journal.pone.0231354 32315342 PMC7173873

[20] Ministry of Health Family Welfare (MOHFW) [Bangladesh]. National neonatal health strategy and guidelines for Bangladesh. Dhaka: MOHFW; 2009.

[21] Rahaman MA, Kalam A, Al-Mamun M. Unplanned urbanization and health risks of Dhaka City in Bangladesh: uncovering the associations between urban environment and public health. Front Public Health. 2023;11:11. doi: 10.3389/fpubh.2023.1269362 PMC10620720 37927876

[22] Lipi AI, Hasan N. Urbanization in Bangladesh: emerging challenges and the way forward. Bangladesh J Multidiscip Sci Res. 2021;3:33–44. doi: 10.46281/bjmsr.v3i1.1112

[23] Islam A, Biswas T. Health system in Bangladesh: challenges and opportunities. Am J Health Res. 2014;2:366–374. doi: 10.11648/j.ajhr.20140206.18

[24] Bishwajit G, Hoque MR, Yaya S. Disparities in the use of mobile phone for seeking childbirth services among women in the urban areas. Bangladesh Urban Health Survey BMC Med Inf And Decis Mak. 2017;17:1–9. doi: 10.1186/s12911-017-0578-2 PMC5747163 29284477

[25] Ahmed S, Creanga AA, Gillespie DG, et al. Economic status, education and empowerment: implications for maternal health service utilization in developing countries. PLOS ONE. 2010;5:e11190. doi: 10.1371/journal.pone.0011190 20585646 PMC2890410

[26] der Heijden J V, Gray N, Stringer B, et al. ‘Working to stay healthy’, health-seeking behaviour in Bangladesh’s urban slums: a qualitative study. BMC Public Health. 2019;19:1–13. doi: 10.1186/s12889-019-6750-0 31101099 PMC6525448

[27] Yaya S, Bishwajit G, Ekholuenetale M, et al. Urban-rural difference in satisfaction with primary healthcare services in Ghana. BMC Health Serv Res. 2017;17:1–9. doi: 10.1186/s12913-017-2745-7 29178876 PMC5702138

[28] Anwar I, Nababan HY, Mostari S, et al. Trends and inequities in use of maternal health care services in Bangladesh, 1991-2011. PLOS ONE. 2015;10:e0120309. doi: 10.1371/journal.pone.0120309 25799500 PMC4370698

[29] Ahmed F, Oni FA, Hossen SS, et al. Does gender inequality matter for access to and utilization of maternal healthcare services in Bangladesh? PLOS ONE. 2021;16:e0257388. doi: 10.1371/journal.pone.0257388 34529701 PMC8445442

[30] Banik BK. Barriers to access in maternal healthcare services in the northern Bangladesh. South East Asia J Public Health. 2016;6:23–36. doi: 10.3329/seajph.v6i2.31832

[31] Henderson C, Dancy MH. Barriers to the use of research-based instructional strategies: the influence of both individual and situational characteristics. Phys Rev ST Phys Educ Res. 2007;3:020102. doi: 10.1103/PhysRevSTPER.3.020102

[32] Ehsan U, Sakib N, Haque MM, et al. Confronting autism in urban Bangladesh: unpacking infrastructural and cultural challenges. EAI Endorsed Trans On Pervasive Health And Technol. 2018;4:e5–e. doi: 10.4108/eai.13-7-2018.155082

[33] Blake JM, Rubenstein E, Tsai P-C, et al. Lessons learned while developing, adapting and implementing a pilot parent-mediated behavioural intervention for children with autism spectrum disorder in rural Bangladesh. Autism. 2017;21:611–621. doi: 10.1177/1362361316683890 28366007

[34] Sori ND. Identifying and classifying slum development stages from spatial data [Master’s Thesis]. The Netherland: University of Twente; 2012.

[35] Hasan SM, Borces KG, Bhattacharyya DS, et al. Healthcare systems strengthening in smaller cities in Bangladesh: geospatial insights from the municipality of Dinajpur. Health Serv Insights. 2020;13:1178632920951586. doi: 10.1177/1178632920951586 32952402 PMC7485152

[36] Agarwal S, Satyavada A, Kaushik S, et al. Urbanization, urban poverty and health of the urban poor: status, challenges and the way forward. Demogr India. 2007;36(1). https://ssrn.com/abstract=3133050

[37] Rodriguez JM, Molnar JJ, Fazio RA, et al. Barriers to adoption of sustainable agriculture practices: change agent perspectives. Renew Agric Food Syst. 2009;24:60–71. doi: 10.1017/S1742170508002421

[38] Akter S, Rahman AKMA. Factors influencing health service utilization among mothers for under-five children: a cross-sectional study in Khulna district of Bangladesh. PLOS ONE. 2022;17:e0274449. doi: 10.1371/journal.pone.0274449 36095009 PMC9467315

