# Supplementary material for: Factors associated with essential newborn care practices among non-institutional births in urban Bangladesh: evidence from Bangladesh Urban Health Survey 2021
Source: Glob Health Action. 2024 Oct 8;17(1):2412152. doi: 10.1080/16549716.2024.2412152 (PMC11463011; doi:10.1080/16549716.2024.2412152)
Supplement: Supplementary table 1.docx [file ZGHA_A_2412152_SM1372.docx]

**Supplementary table 1: Questions asked of the respondents to collect data on essential newborn care practices**

| **Name of ENC practices components** | **Questions asked to collect data for ENC practices** | **Response options** |
| --- | --- | --- |
| Utilization of boiled instruments before cutting the umbilical cord | Was the instruments (Blade, Bamboo strips, Scissor, and others) boiled before the cord was cut? | Yes (1)  No (2)  Don’t know (8) |
| Umbilical cord care that applied of any substances after it was cut and tied | Was anything (antibiotics, antiseptic, spirit/alcohol, mustard oil with garlic, chewed rice, turmeric juice/powder, ginger juice, shidur, boric powder, gentian violet, talcom powder, ash, and others) applied to the cord immediately after cutting and tying it? | Yes (1)  No (2)  Don’t know (8) |
| Drying the newborn less than 5 minutes of birth | How long after birth was (NAME) dried? | <5 minutes (1)  5-9 minutes (2)  10+ minutes (3)  Not dried (4)  Don’t know (8) |
| Wrapping the newborn less than 5 minutes of birth | How long after birth was (NAME) wrapped? | <5 minutes (1)  5-9 minutes (2)  10+ minutes (3)  Not wrapped (4)  Don’t know (8) |
| Delayed bathing (72+ hours after birth) | How long after delivery was (NAME) bathed for the first time? (If less than one hour recorded in “00”, less than one day recorded in hours, and less than one week recorded in days). | Hours (1)  Days (2)  Weeks (3)  Not bathed (995)  Don’t know (998) |
| Initiation of breastfeeding within 1 hour of delivery | How long after birth did you first put (NAME) to the breast? (If less than 1 hour recorded ‘00' hours, less than 24 hours recorded in hours, otherwise recorded in days). | Immediately (00)  Hours (1)  Days (2) |
